# Status and strategies of college English teaching using adaptive deep learning from the perspective of multiculturalism

**DOI:** 10.3389/fpsyg.2022.910667

**Published:** 2022-07-28

**Authors:** Bi Fei, Xia Cai, Guo Huang

**Affiliations:** ^1^Institute of Education, Sichuan Normal University, Chengdu, China; ^2^College of Foreign Languages and Cultures, Chengdu University of Technology, Chengdu, China; ^3^Sports Institute, Chengdu University of Technology, Chengdu, China; ^4^Sichuan Province University Key Laboratory of Internet Natural Language Intelligent Processing, Leshan Normal University, Leshan, China

**Keywords:** adaptive deep learning, college English, edge computing, strategy research, English teaching

## Abstract

The aim is to deeply understand the current situation of College English Teaching (CET). By consulting the theories of Deep Learning (DL) and Edge Computing (EC), this work designs a Questionnaire Survey (QS) to understand the current situation of college English learning and teaching. Then, Adaptive Deep Learning (ADL) and EC are introduced into CET. Finally, the corresponding conclusions and suggestions are drawn. Specifically, the purpose and time of college students’ vocabulary learning are investigated. The results suggest that students’ English vocabulary learning is shallow. They have not really mastered the meaning and usage of vocabulary. Simultaneously, teachers’ vocabulary teaching methods are analyzed from three aspects: root affixes, vocabulary collocation, or repeated reading and memory. The teaching method is excellent from the teacher’s perspective but far from perfect from the students’ perspective. Introducing ADL and EC into CET enhances students’ class concentration time, homework submission efficiency, and academic performance. More than half of the students believe that the new teaching method introducing ADL and EC can make them more confident and motivated in English learning. Therefore, it has great reference significance for applying ADL and EC in CET.

## Introduction

### Research background

English is one of the most widely used languages in the world and plays an increasingly important role in interpersonal communication. In China, English is now one of the compulsory subjects for students (Jin et al., 2019). Multiculturalism is the result of the complex human society driven by an ever-frequent information circulation and the constant renewal and transformation of culture. Under multiculturalism, various cultures face new opportunities and challenges, and original cultural phenomena emerge. Deep Learning (DL) is the internal law and representation level of learning sample data. The information obtained in the learning process is beneficial to interpreting data, such as text, images, and sound. Therefore, from the perspective of multiculturalism, this work introduces the Adaptive Deep Learning (ADL) theory into College English Teaching (CET). It explores whether this theory can strengthen students’ understanding of English learning.

### Literature review of English teaching

Qiu (2019) believed that globalization provided rich resources for English expression but made traditional discourse break away from the progress of the times and failed in actual discourse experiments. The result was the discourse failure dilemma (Qiu, 2019). Peter (2018) analyzed the learning motivation and strategies of college students based on cognitive psychology and divided learning methods into shallow learning and DL (Peter, 2018). Through experimental research, Parsons (2019) proved that the ADL-based method could improve students’ critical thinking ability. They found that DL methods could help students process knowledge information reasonably and scientifically and rapidly integrate information (Parsons, 2019). Jaime (2020) proposed that applying Edge Computing (EC) and ADL in CET was becoming a reality. For example, relevant researchers studied applying assisted instruction, detecting users’ behavioral attitudes in various ways, and analyzing students’ learning status (Jaime, 2020). Compared with the research in this work, Jaime’s (2020) research has a certain one-sidedness: it only considers the topic from the perspective of user behavior posture. After analyzing the current situation of CET, this work puts forward valuable strategies, thus presenting some systematicness. Dai (2019) proposed an idea of English teaching construction based on EC and ADL, which contributes to the construction of English teaching (Dai, 2019). The above scholars have elaborated their views and methods on English learning from different perspectives but have not yet put forward practical suggestions and measures for CET in the future. In contrast, after analyzing the data results, this work optimizes the future English teaching path in Colleges and Universities (CAUs) and puts forward corresponding suggestions.

### Literature review on teaching vocabulary

Mandalorian (2018) reasoned that although many scholars studied vocabulary ability, the attention to high school students was not enough. There were only some studies on vocabulary teaching and learning for high school students. There was still room for detailed research in vocabulary teaching (Mandalorian, 2018). Saussure (2020) studied the relationship between vocabulary and grammar and proposed the lexical chunk teaching method (Saussure, 2020). Li (2018) found that activation diffusion could model the mental vocabulary organization into an interconnected spider web. The words on the web had networked features like relationship (association). These associations could express the meaning of particular vocabularies (Li, 2018). The above scholars put forward their views and methods from the vocabulary teaching perspective. By comparison, from the overall point of view, this paper mainly analyzes and discusses the current teaching situation and less involves the vocabulary part.

### Research meaning

There are differences between this work and the previous studies. In the previous studies, the ideas of English teaching construction, the learning motivation and strategies of college students, and the effect of ADL on learning have been elaborated from different angles. However, no practical suggestions and measures have been put forward for the future CET. In view of this, this work attempts to understand the current situation of CET through the Questionnaire Survey (QS) method from the perspective of ADL and multiculturalism. Then, it introduces the ADL and EC methods into college English classrooms and analyzes the impact of the proposed method on students’ English learning. Finally, valuable suggestions are formulated. It aims to provide a methodological reference for further in-depth analysis of the current situation of CET.

## Theory and method

### Construction of a DL model by CET strategies

**Figure 1:**
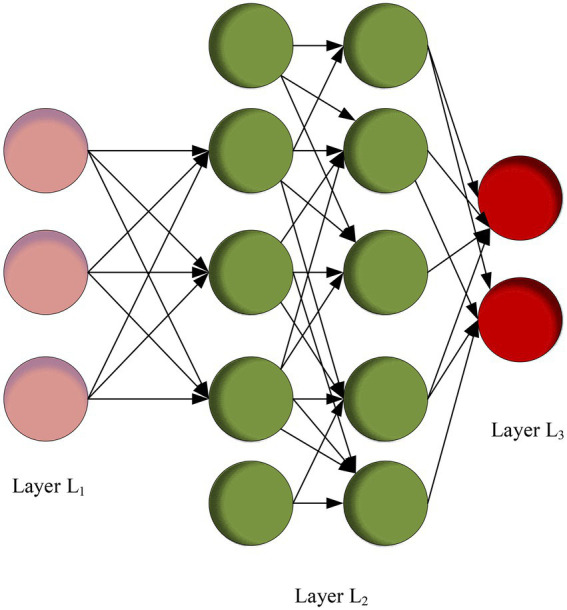
DL model.

DL is the currently most-pursued Machine Learning (ML) method. ML studies computer simulations or the realization of animal learning behaviors. Through ML, new knowledge or skills can be learned, existing data structures can be rewritten, and program performance can be improved (Disogra, 2019). DL has a strong correlation with the Neural Network (NN) models in ML since DL relies mainly on the NN backbone to implement. Thus, DL is sometimes called the improved NN algorithm (Zhang et al., 2019; Breden and Moore, 2020). The theoretical model of DL is shown in Figure 1.

DL algorithm can correct the weights and deviations of the neuron layer by observing the error function gradient descent (Walsh, 2019). The calculation method is shown in Equation (1).


(1) $$Q_{a+1}=Q_{a}-c_{a}d_{a}$$

In Equation (1), $Q_{a+1}$ represents the weight and deviation of the neuron layer, $Q_{a}$ is the weight and deviation after iterative calculation, $c_{a}$ denotes the learning rate of the NN, and $d_{a}$ signifies the gradient of the error function.

In addition, the specific calculation of the space size of the dilated convolution is given in Equation (2).


(2) $$A=\left(n-1\right)*b+1$$

In Equation (2), A represents the size of the convolution kernel space, n is the size of the original convolution kernel, and b denotes the void ratio. The calculation of the image size after convolution reads:


(3) $$B=\left(l-K+2P\right)/S+1$$

In Equation (3), B is the image size after convolution, l represents the input image size, K denotes the output image size, P means the padding size, S stands for the step size.

Non-negative Matrix Factorization (NMF) is an effective DL method to find more non-negative matrices to make the final product as close to the original matrix as possible. It realizes the non-linear dimensionality reduction of the data (Wu et al., 2020; Shafie et al., 2021). The calculation of the norm cost function by the matrix difference is expressed in Equation (4).


(4) $$\min_{P,W,Q}||Y-PWQ^{T}||{}_{F}^{2}$$

In Equation (4), $Q^{T}$ represents the transposition of the matrix Q and $._{F}$ is the norm of the matrix difference. P,W,Q,Y denote different matrices. The updated decomposition calculation method is listed in Equations (5–7).


(5) $$P_{ij}\leftarrow P_{ij}\frac{\left({YQW}^{T}\right)}{{\left({PWQ}^{T}{QW}^{T}\right)}_{ij}}$$

(6) $$W_{ij}\leftarrow W_{ij}\frac{{\left(P^{T}YQ\right)}_{ij}}{{\left(P^{T}{PWQ}^{T}Q\right)}_{ij}}$$

(7) $$Q_{ij}\leftarrow Q_{ij}\frac{{\left(YPW\right)}_{ij}}{{\left({QW}^{T}P^{T}PW\right)}_{ij}}$$

$P_{ij}$ represents the element in the i-th row and j-th column of the matrix P. $W_{ij}$ is the element in the i-th row and j-th column of the matrix W. $Q_{ij}$ denotes the element in the i-th row and j-th column of the matrix Q. P,W,Q,Y mean different matrices. $W^{T},\;Q^{T},\;P^{T}$ stand for the transpose of different matrices.

Multi-Task Learning (MTL) is an important part of the DL method. MTL can solve the DL data segmentation problem by finding useful information from related data (Stella et al., 2019; Tai et al., 2019; Mikrani et al., 2020). According to the definition, the calculation of the MTL model reads:


(8) $$\min\overset{M}{\underset{m}{\sum }}L\left(X^{m},Y^{m},W^{m}\right)+\lambda Reg\left(W\right)$$

In Equation (8), $X^{m},\;Y^{m},\;W^{m}$ represent the input X,Y,W matrix of the m-th task, M is the total number of samples, and Reg denotes the regularization constraint. λ refers to the weight of control regularization constraints.

Next, to ensure research data accuracy, the Accuracy and Recall need to be measured by Equations (9, 10).


(9) $$\mathrm{Accuracy}=\frac{TP+TN}{P+N}$$

(10) $$Re\mathrm{call}=\frac{TP}{TP+FN}=\frac{TP}{P}$$

TP represents the number of positive examples that are correctly divided. TN is the number of negative examples that are correctly classified P is TP+FN, where FN means the number of negative examples that are incorrectly divided, and N signifies the total number.

DL is a general term for a class of Pattern Analysis (PA) methods. Here, DL is introduced into CET to reconstruct students’ problems and doubts in the learning process. In this way, students can conduct research and exploration based on previous concepts. Additionally, introducing DL in CET can also form a complete set of learning logic strategies: knowledge construction - > problem raising - > problem-solving - > learning effect evaluation. After consolidating and reviewing the learned knowledge, students can deepen their initial understanding of learning and find joy in learning. After analyzing the research content of DL, three different methods are mainly involved.

**Figure 3:**
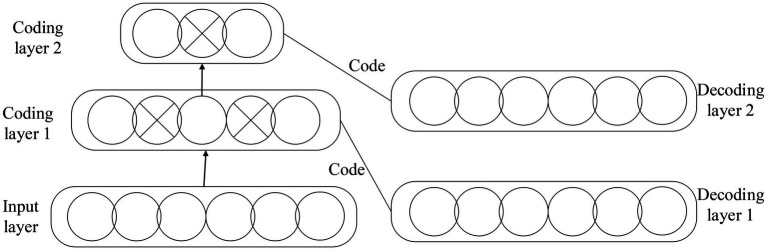
AE NN model.

**Figure 4:**
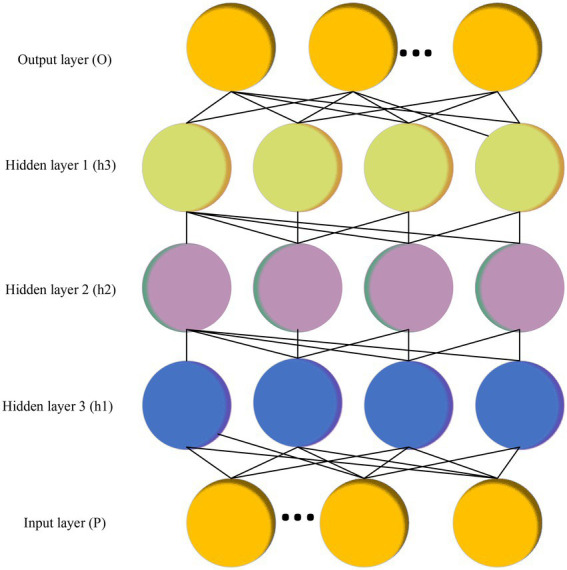
DBN Model (O represents the output layer. h1, h2, and h3 are the different weights of the hidden layer. P denotes the input layer).

1. Convolutional Neural Networks (CNN). CNN uses convolution operations and includes a deep feedforward NN with input, convolution, pooling, and fully connected layers. CNN is one of the representative algorithms of DL (Ilse et al., 2019; Tian et al., 2019). A typical CNN structure is depicted in Figure 2.
  **Figure 2:**
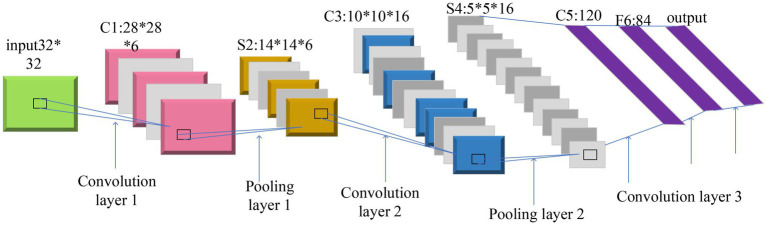
CNN model.
  The calculation method is shown in Equations 11–15.
(11) $$Rt=ℑ\left(B_{t}\left[b_{t-1},\;x_{t}\right]+P_{f}\right)$$
(12) $$Y_{t}=ℑ\left(A_{I}\cdot \left[b_{t-1},\;x_{t}\right]+P_{I}\right)$$
(13) $$Z_{t}=\tan b\left(B_{D}\cdot \left[b_{t-1},x_{t}\right]+C_{D}\right)$$
(14) $$D_{t}=T_{t}\times D_{t-1}+I_{t}\times D_{t}$$
(15) $$E_{t}=\partial \left(A_{E}\left[b_{t-1},\;x_{t}\right]+C_{E}\right)$$
  In Equations (11–15), Rt represents the forget gate. ℑ is the hidden layer neuron. $B_{t}$ denotes the model output. $b_{t-1}$ indicates the hidden layer information at t−1 moment. $x_{t}$ means the input at t moment. $Y_{t}$ signifies the input gate. $P_{f}$ and $P_{I}$ are the Activation Function (AF) of the forget gate and the input gate, respectively. $Z_{t}$ stands for the candidate memory cell information. b describes the function coefficient. $B_{D}$ explains the bias of the convolution kernel. $C_{D}$ illustrates the maximization pool. $T_{t}$ signals the average pooling. $D_{t-1}$ demonstrates the hidden layer of the candidate’s memory information at this moment. $E_{t}$ implies the output gate. $A_{E}$ refers to the probability obtained by the function. $C_{E}$ presents the word vector dimension.
2. The Auto Encoder (AE) NN with multi-layer neurons include Auto Encoder and Sparse Coding (Li et al., 2019; Yuan and Wu, 2019), as detailed in Figure 3.
3. The multi-layer AE NN is pre-trained. Then, the identification information is used to optimize the network weights so that the Deep Belief Network (DBN) model can be used for unsupervised learning or supervised learning. Figure 4 gives the details.

**Figure 5:**
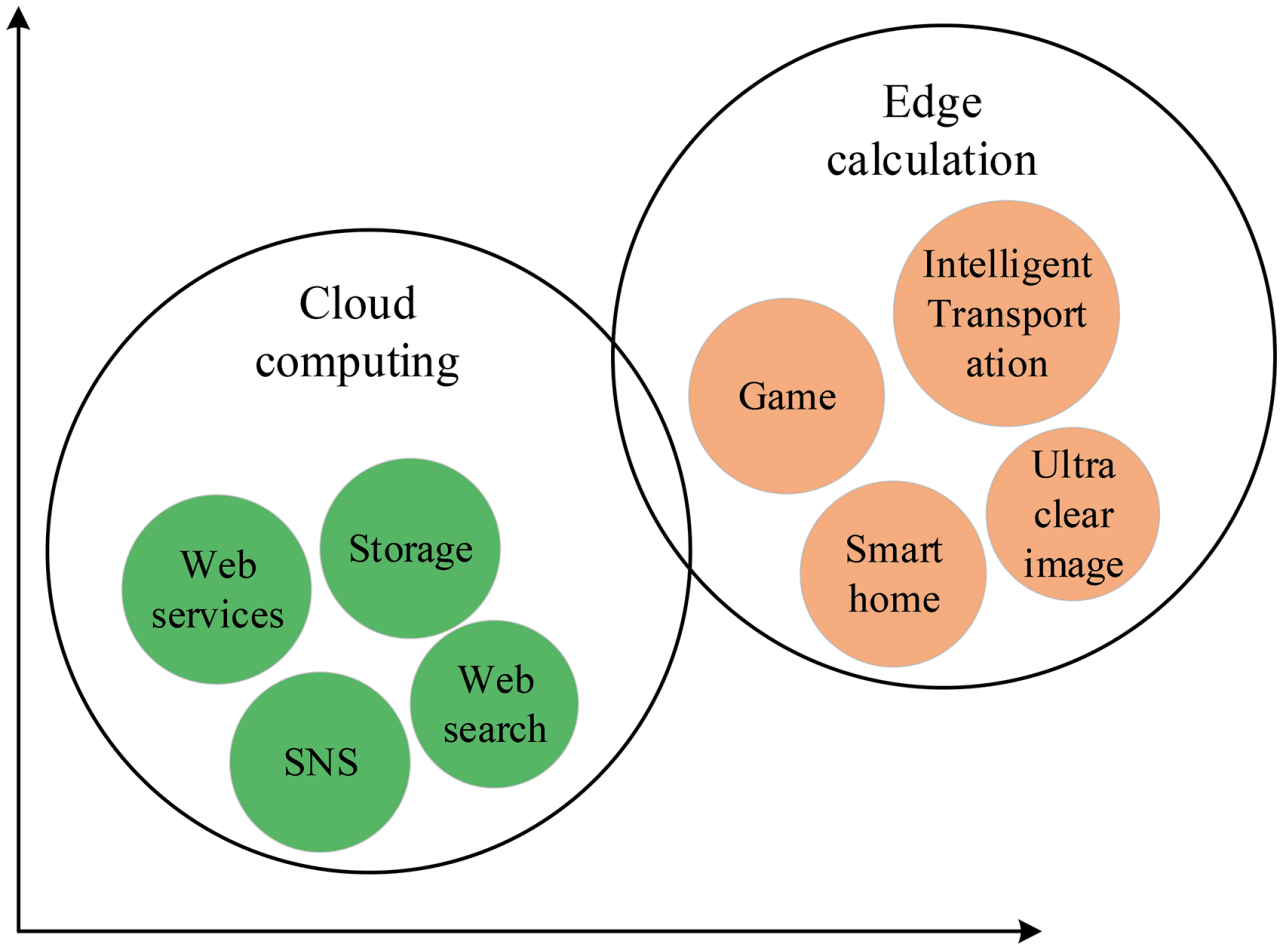
Differences between CC and EC.

EC refers to an open platform that integrates network, computing, storage, and application core capabilities on the side close to the object or data source. It can provide the nearest-end services (Liu et al., 2019a,b). Meanwhile, CET can integrate DL and EC. The DL method-generated data must use Artificial Intelligence (AI) technologies to release their value and potential fully. EC can flourish with richer data and application scenarios (Majumdar and Pal, 2020). Combining EC and DL can shorten the data transmission time and improve security performance. The advent of the “Internet +” era has provided an opportunity for the rapid development of Mobile Intelligence (MI) and EC (Han et al., 2019; Modgill et al., 2020). In particular, EC is a technology developed under the background of high bandwidth, time-sensitive, and integrated Internet of Things (IoT), much different from Cloud Computing (CC). Figure 5 compares EC and CC specifically.

The data to be processed will be sent to the computer cloud. The EC time consumption is calculated by Equation (16).


(16) $$T_{\mathrm{cloud}}=T_{\mathrm{transmission}}+T_{\mathrm{propagation}}+T_{c\mathrm{Pr}oc}+T_{\mathrm{queue}}$$

In Equation (16), the EC time consumption in the computer cloud is the sum of propagation, transmission, processing, and queuing delays. Of these, transmission delay accounts for the most. Meanwhile, EC time consumption is also affected by the speed of the wireless network. Therefore, the data transmission delay calculation reads:


(17) $$T_{\mathrm{transmission}}=\max\left(\max_{z=1,\;2\ldots n}\left(s_{i}/b_{i}\right)\right)$$

In Equation (17), $s_{i}$ represents the data collection speed, and b is the outlet bandwidth of each network node. The propagation delay is the data propagation speed per unit distance, as counted in Equation (18).


(18) $$T_{\mathrm{propagation}}=d/v$$

In Equation (18), d represents the average distance from the data source to the cloud, and v denotes the propagation speed of light. Then, the EC time consumption is expressed by Equation (19).


(19) $$T_{\mathrm{edge}}=T_{e\mathrm{Pr}oc}+T_{\mathrm{control}}$$

In Equation (19), the EC time consumption is the sum of the processing delay and the edge network controller’s time executing the scheduling algorithm.

EC is a subcategory of CC, and their relationship is depicted in Equation (20).


(20) $$y_{\mathrm{cloud}}=\partial y_{\mathrm{edge}}$$

In Equation (20), ∂ represents a constant >1. Obviously, the power of cloud computing is ∂-th that of EC.

### Research methods to be adopted

(1) Case study: By collecting relevant data, experts’ and scholars’ views are cited in the case study on CET strategies from the perspective of multiculturalism. Based on the various problems and phenomena in the current social reality, the corresponding research framework has been established to make this work more scientific (Wang et al., 2020). (2) Comparative analysis method uses the multi-party comparison of two or more research objects to discover their similarities and differences. Then, the most suitable methods are analyzed and used for reference to provide strategies for CET (Kertys et al., 2020). (3) Quantitative and qualitative analysis method researches and summarizes the characteristics of quantity, the logical relationship between quantities, and the trend of quantity changes by collecting relevant research data. In qualitative analysis, forecasters analyze the future development trend and nature of data using relevant historical data, government policies, and major events in society (Xavier et al., 2020). (4) In a QS, researchers use controlled measurement to measure the problems studied to collect reliable data. In order to understand the current situation of English teaching in Chinese CAUs, 300 QSs are randomly distributed to college teachers and students in December 2019 and May 2020. For the scientificity concern, the QS contents are discussed with experts of relevant majors before issuing and modified accordingly. For a higher recovery rate, face-to-face distribution and on-the-spot recovery are chosen to recover 270 copies, with 239 effective ones, reaching a 90% recovery rate and an effective recovery rate of about 88.52%. The characteristics of the respondents are shown in Table 1.

**Table 1:** Characteristics of respondents.

Type	Detailed	Number of people	Proportion(%)
Gender	Male	120	50.2%
	Female	119	49.8%
Grade	Freshman	60	25.1%
	Sophomore	60	25.1%
	Junior	60	25.1%
	Senior	59	24.7%
English background	Below level 4	34	14.23%
	English Band 4	154	64.4%
	English Band 6	46	19.25%
	TOEFL / IELTS	5	2.12%

The Kaiser-Meyer-Olkin (KMO) coefficient is introduced to verify the designed QS and calculated by Equation (21).


(21) $$KMO=\frac{\sum \sum_{i\neq j}r_{ij}^{2}}{\sum \sum_{i\neq j}r_{ij}^{2}+\sum_{i\neq j}r_{ij•1,2\ldots k}^{2}}$$

In Equation (21), r represents the correlation coefficient, i is the dependent variable, j denotes the independent variable, and k indicates the quantity. KMO measurement standards are listed in Table 2.

**Table 2:** KMO measurement standards.

Type	Range of values	Is it for factor analysis?
KMO value	<0.9	Very much suitable
	0.8 ~ 0.9	Very suitable
	0.7 ~ 0.8	Fit
	0.6 ~ 0.7	Not very suitable
	0.5 ~ 0.6	Barely fit
	>0.5	Unsuited

Next, with reference to statistical knowledge, the QS validity is verified by SPSS 23. As a result, KMO = 0.869, between 0.8 and 0.9; *p* = 0, less than 0.01. Therefore, the obtained data are suitable for factor analysis, and the designed QS has good validity.

## Results and analysis of CET introducing DL and EC

### The current situation of CET from the perspective of multiculturalism

**Figure 6:**
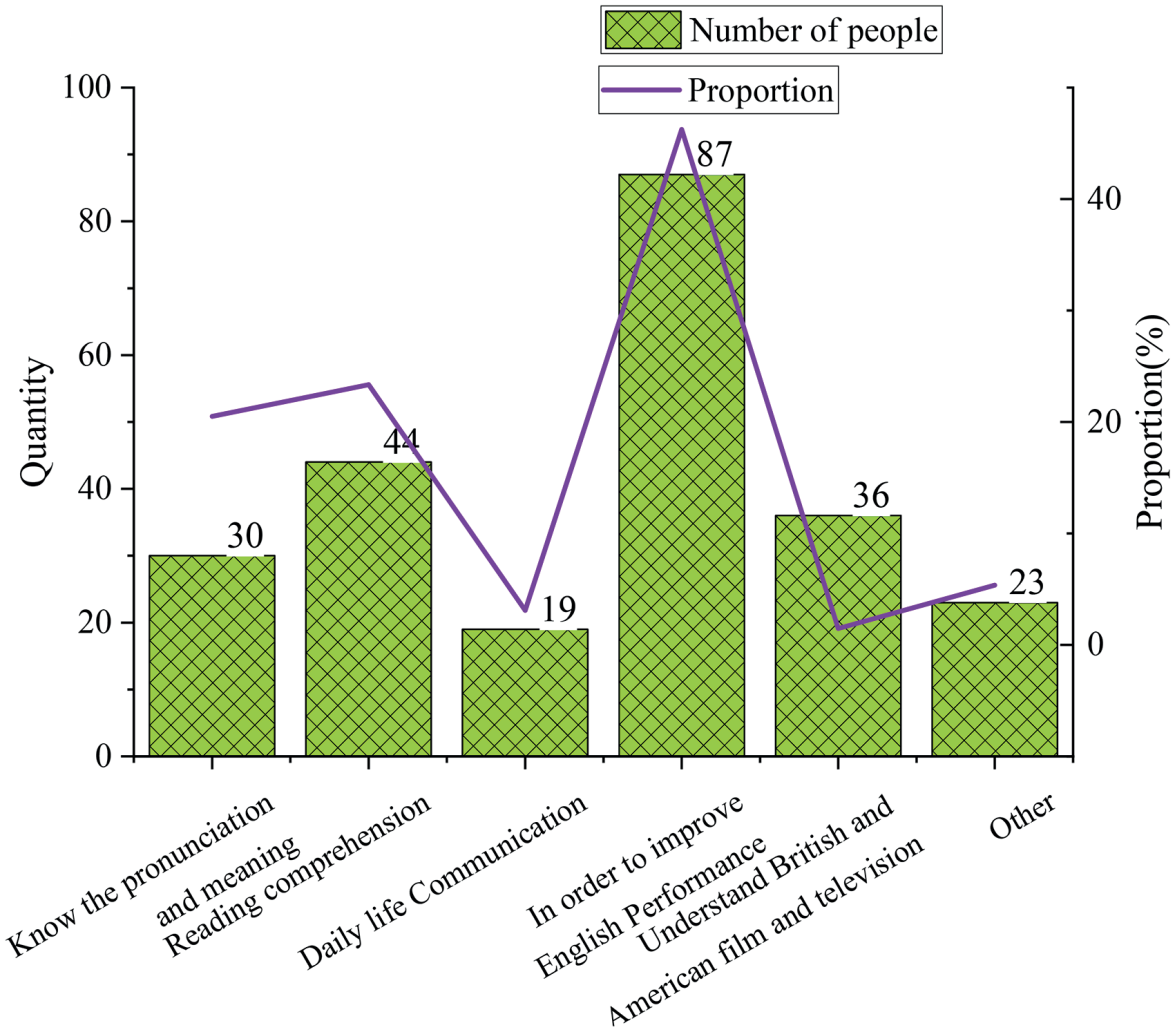
The purpose of learning English vocabulary for college students.

This section understands the current situation of CET by considering students’ English vocabulary learning. According to the QS result, the purpose of vocabulary learning for college students has five aspects, as detailed in Figure 6.

According to Figure 6, college students learn English vocabulary to: (1) Know the basic pronunciation and meaning; (2) Improve the English Proficiency Level (EPL) in reading comprehension; (3) Communicate with English in daily life; (4) Improve English performance; and (5) Understand British and American film and television. Of these, most respondents aim to improve English performance, accounting for 46.25% of the total. The second most respondents learn English vocabulary to improve reading comprehension, accounting for 23.34%. Then, the respondents aim to know the basic pronunciation and meaning account for 20.51%. Finally, those who want to understand British and American film and television and use it for daily communication account for 1.47 and 3.1%, respectively. Thus, college students’ primary goal in learning English is academic achievement.

**Figure 7:**
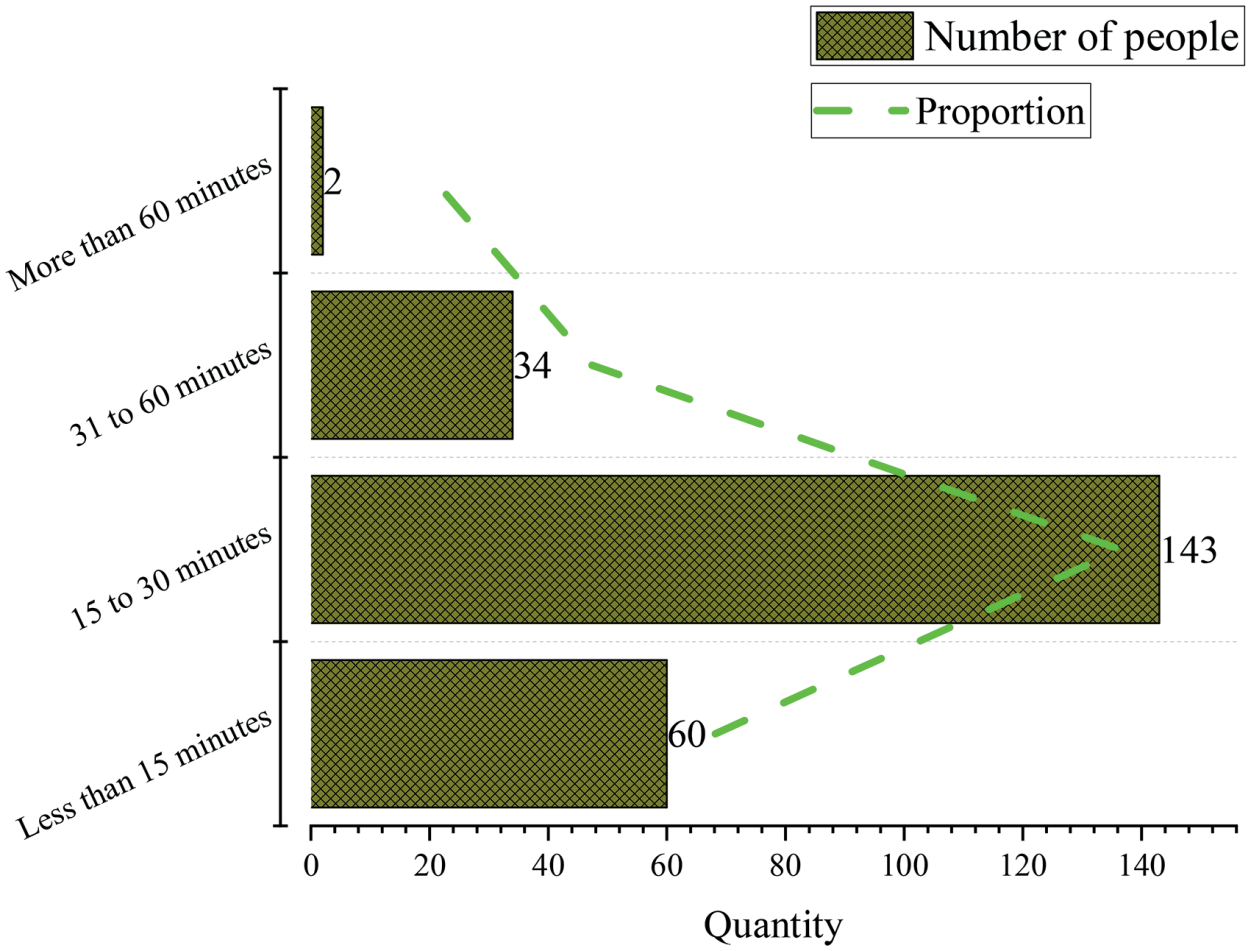
Time spent by college students learning English vocabulary.

Subsequently, Figure 7 summarizes college students’ time learning English vocabulary every day.

Apparently, 24.95% of respondents plan to learn vocabulary for less than 15 min every day, 60.03% of the students learn vocabulary for 15–30 min, 14.31% of students learn vocabulary for 31–60 min, and 0.71% of students learn vocabulary for more than 60 min. Thus, most respondents spend between 15–30 min learning vocabulary, followed by those who spend less than 15 min. Very few students spend over 60 min. Surely, the lack of investment in vocabulary time will lead to the decline of the learning effect.

**Figure 8:**
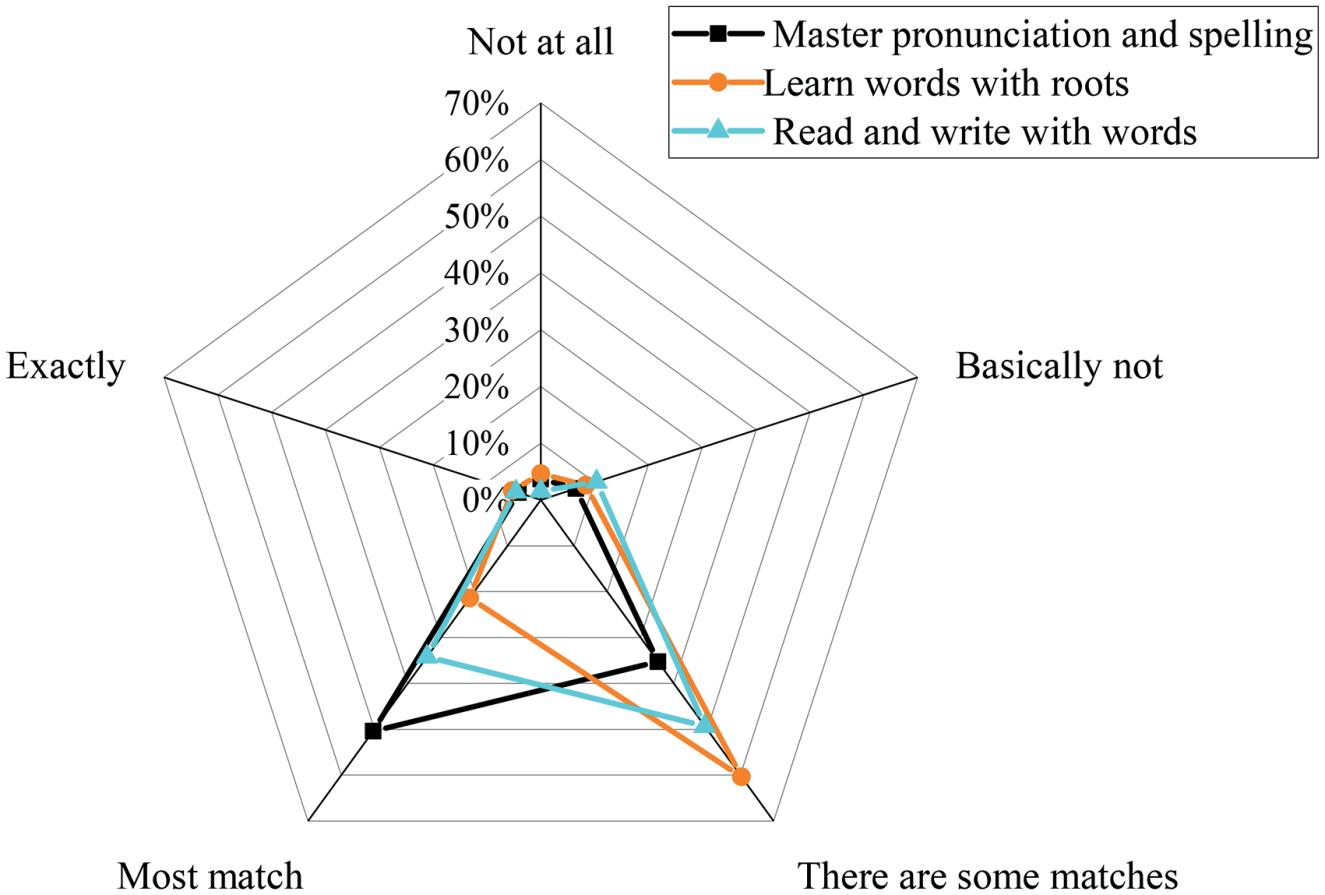
The mastery of the basic connotation of English vocabulary by college students.

Figure 8 plots the specific results of students’ mastery of the basic connotations of English vocabulary.

Vocabulary is the most basic part of English learning. From Figure 8, in terms of mastering pronunciation, spelling, and basic meaning, 50.39 and 35.24% of respondents either have mastered most knowledge points or part of knowledge points. Very few respondents have fully mastered the knowledge points or have no mastery at all. This might result from most students’ mechanical memorization without deeply thinking about the meaning of the vocabulary. There is a severe lack of standardized learning: many students still have prominent problems with sound, form, and meaning. They only have a general grasp of the basic connotation of words. When students learn new words using word roots, sounds, and forms based on their previous knowledge, 60.34% of the respondents can generally perform right. 21.36% of the respondents can perform mostly right. The rest of the respondents either perform perfectly or show a total disability. Hence, there are still problems in connecting college students’ learning and cognition of new words. Finally, in terms of using the learned words for reading and writing, about 49.29% of the respondents can perform generally well. About 34.16% of the respondents perform mostly well. Very few respondents can perform perfectly well or show a total disability. Therefore, college students also have problems when using new words in practical applications. Probably, the usage frequency of English is not high, and some students forget as they memorize. Additionally, the ability to flexibly use words is not enough, and expanding old words into new linguistic scenarios has not been realized.

**Figure 9:**
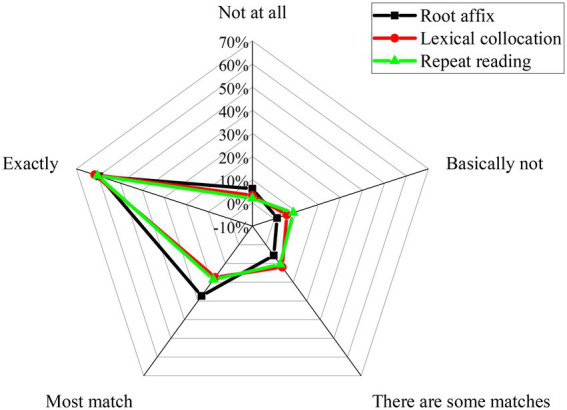
The way college teachers teach vocabulary.

Teachers are the mainstay of CET. Thus, the way teachers teach vocabulary is understood and analyzed in Figure 9.

Figure 9 implies that college teachers’ vocabulary teaching methods mainly focus on “using root affixes to explain words (Scenario 1), using word collocation to explain words (Scenario 2), and having students memorize words by repeating (Scenario 3).” In Scenario 1, 59.56% of the respondents can accept perfectly well. 27.36% of respondents can accept mostly well. In Scenario 2, about 61.66% of respondents can accept perfectly well. 17.43% of respondents can accept mostly well. By contrast, in Scenario 3, about 60.39% of respondents can accept perfectly well, and 18.59% of respondents can accept mostly well. Thus, three scenarios of vocabulary teaching methods can basically meet students’ requirements, which is beneficial to students from teachers’ perspectives. However, the result might not be completely consistent with the above QS results of students. That is to say, college teachers should make full use of these vocabulary teaching methods. Moreover, they should follow the development trend of the times, constantly introduce new skills and methods, and innovate teaching strategies and skills.

### Application of DL and EC in CET

**Figure 10:**
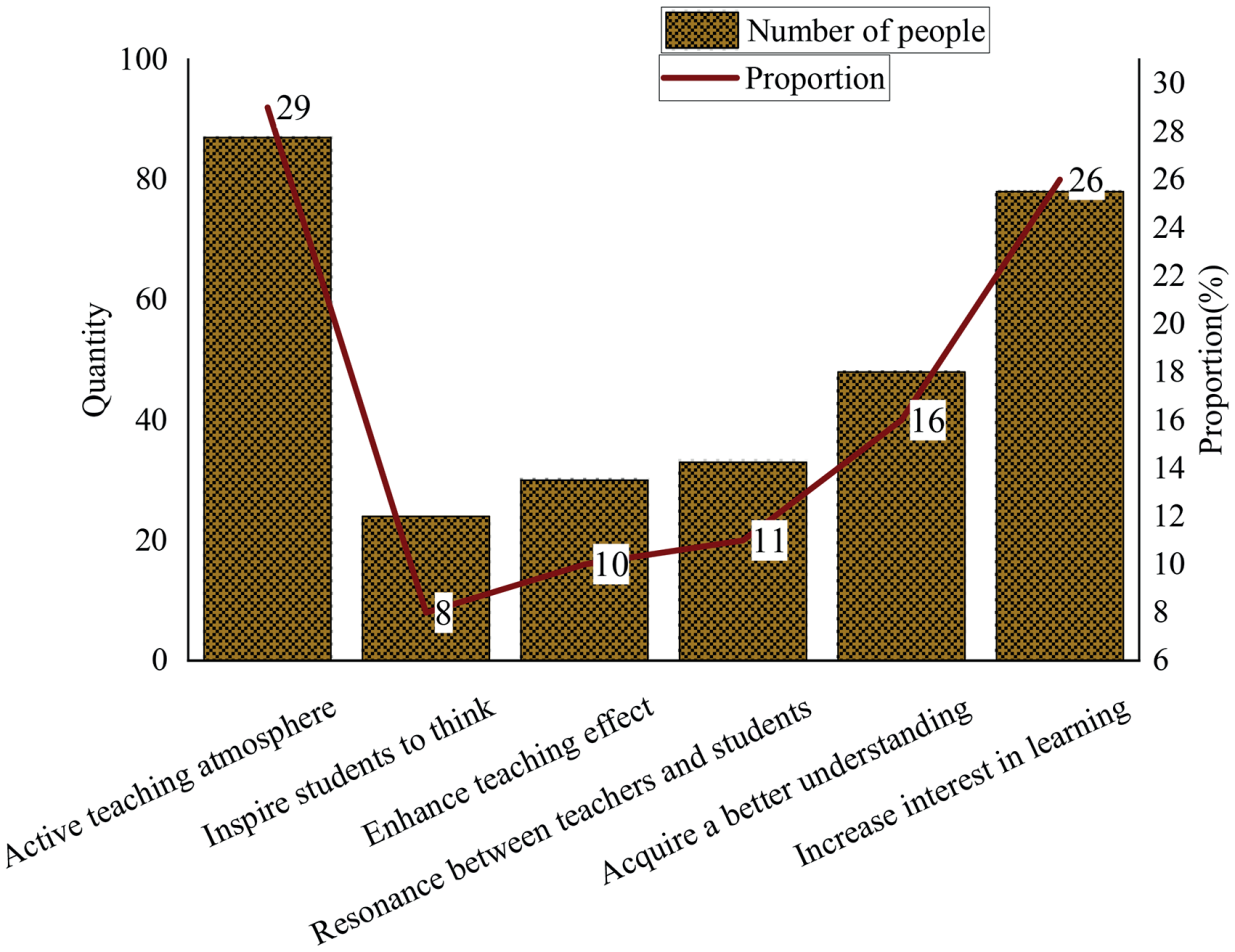
The advantages of introducing DL in CET over conventional teaching.

This section tries to solve the problems found in the QS by integrating ADL and EC methods into CET. Three hundred students are interviewed. The advantages of introducing ADL and EC methods in CET over conventional teaching are summarized in Figure 10.

From Figure 10, most respondents believe that ADL and EC methods can activate the teaching atmosphere and make the classroom livelier. Another 11% of respondents believe that it can make students and teachers interact and resonate and enhance the relationship between teachers and students. Another 10% of the respondents think it can enhance the teaching effect of teachers and arouse students’ willingness to take the course. Another 26% of respondents think it can attract students’ interest in learning to help them engage in classroom activities more actively. Another 16% of the respondents believe it helps them deepen their understanding of English vocabulary and easily absorb and remember knowledge points. Finally, 8% of the respondent believe that ADL and EC methods can inspire students’ thinking to avoid mind slips. Therefore, introducing ADL and EC into CET has been welcomed by most respondents and is the right decision.

The 300 students are randomly divided into two groups. The specific results are shown in Table 3.

**Table 3:** Number and proportion of respondents.

Group	People number	Proportion
Group one	150	50%
Group two	150	50%

**Figure 11:**
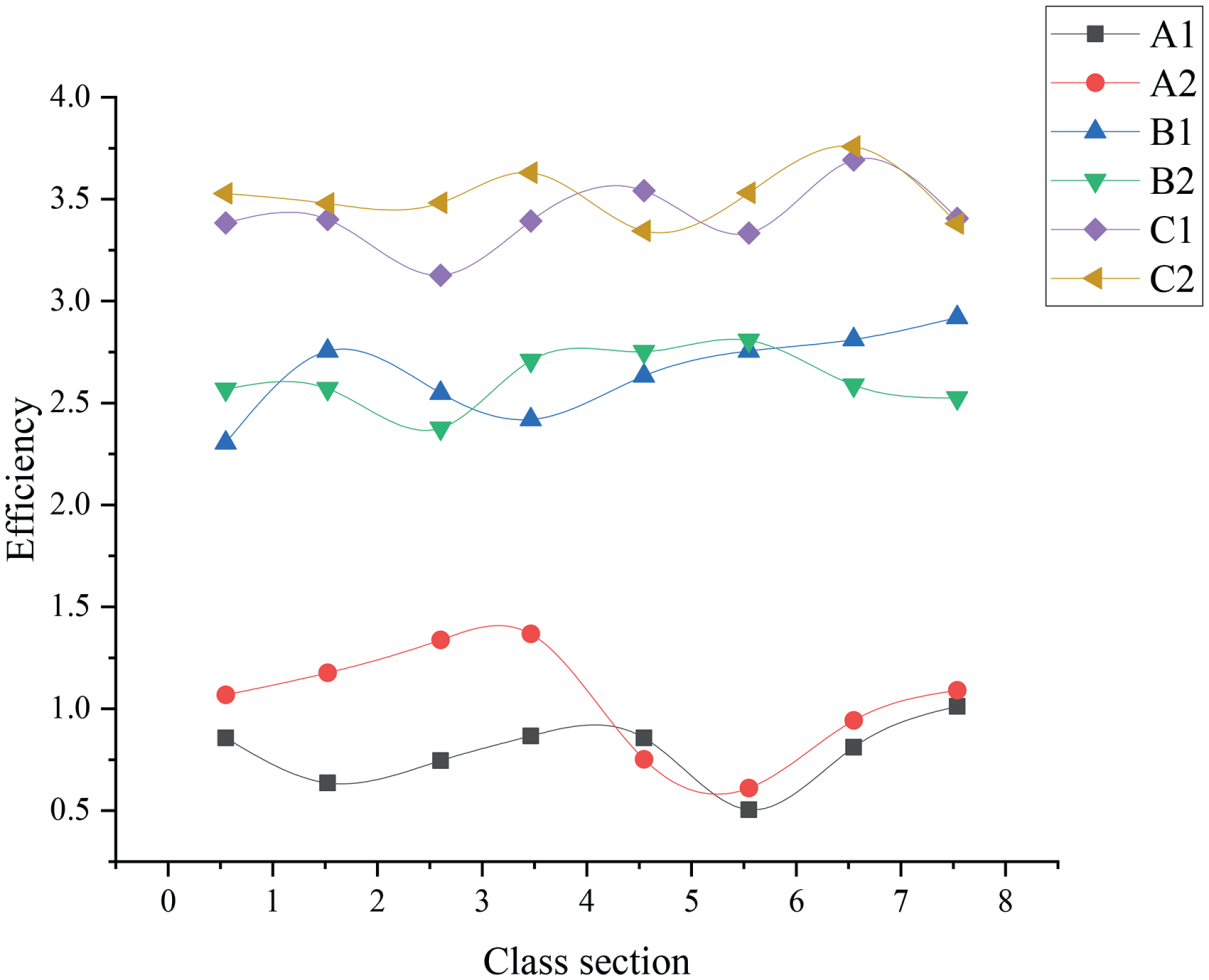
Student work efficiency before the experiment (A1 means ordinary student in Group 1, A2 means ordinary student in Group 2, B1 above-average student in Group 1, B2 means above-average student in Group 2, C1 means extraordinary student in Group 1, C2 means extraordinary student in Group 2).

**Figure 12:**
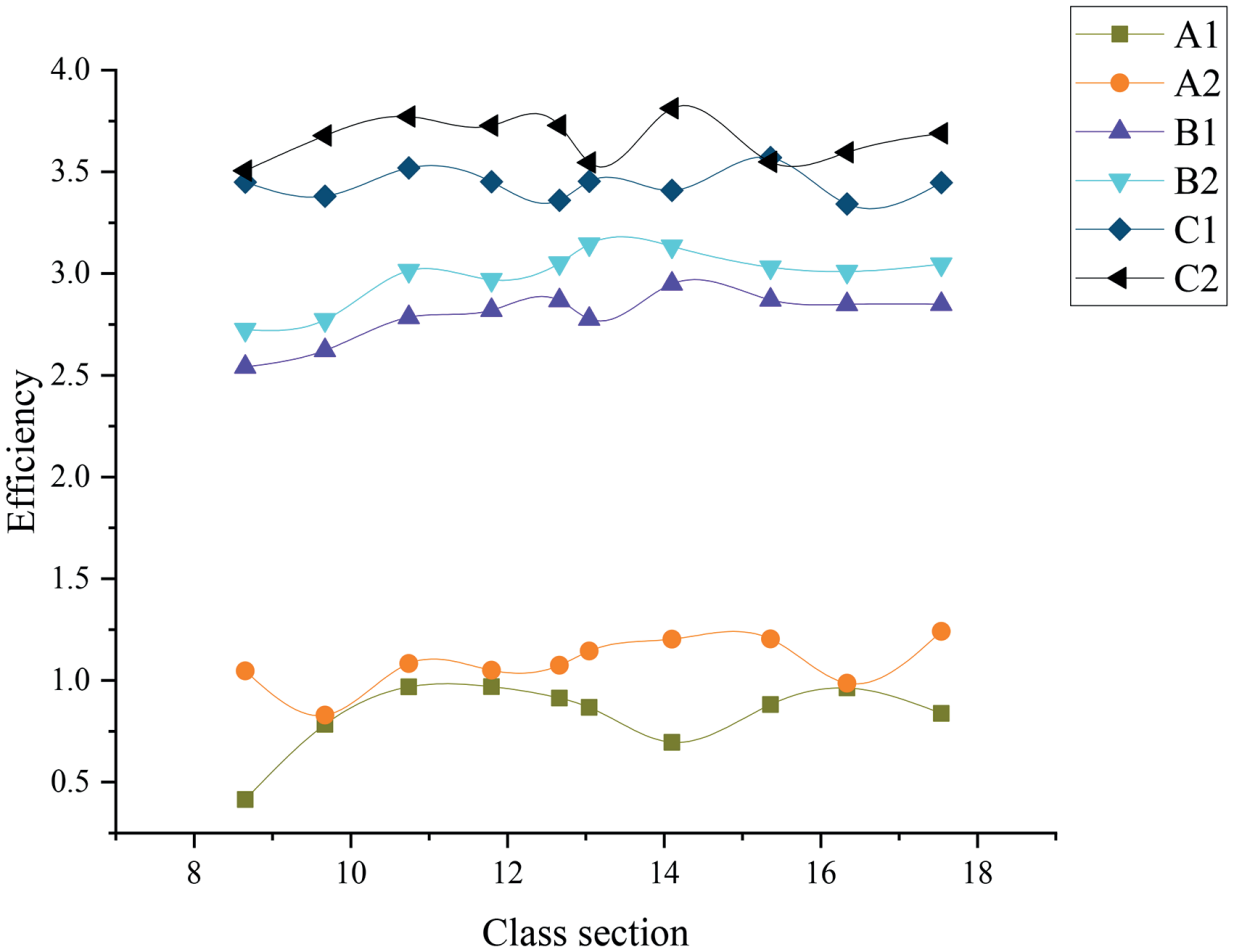
The work efficiency of students after the experiment (A1 means ordinary student in Group 1, A2 means ordinary student in Group 2, B1 above-average student in Group1, B2 means above-average student in Group 2, C1 means extraordinary student in Group 1, C2 means extraordinary student in Group 2).

Figures 11, 12 examine the impact of ADL and EC methods on CET by factoring in students’ homework performance.

Obviously, introducing ADL and EC methods has not significantly changed the work efficiency of extraordinary students whose performance tends to be stable. Thus, the experiment still has a relatively small impact on the efficiency of the extraordinary students. By comparison, the work efficiency of ordinary students has changed to a certain extent, especially those in Group 2 who have changed significantly. The work efficiency of above-average students has been greatly improved, and they gradually approached the extraordinary students. Therefore, after introducing ADL and EC, the impact on above-average students has been further improved after teaching strategy adjustment. Although there is still a gap, the gap between the ordinary and above-average students after the experiment has reduced significantly. Hence, ordinary students’ learning attitude has improved after the experiment: the teaching strategy adjustment by introducing ADL and EC has positively impacted ordinary students.

**Figure 13:**
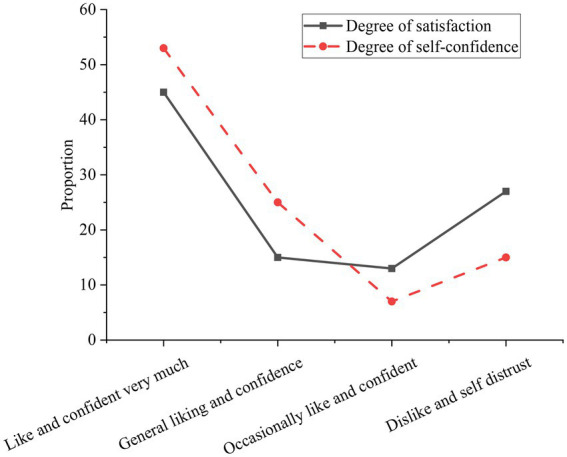
Evaluation of college students’ experience of introducing ADL and EC methods.

Additionally, students’ usage experience is investigated in Figure 13.

In Figure 13, the students who like the new learning method account for about 72%. About 13% of students do not like the new learning method. 15% of students sometimes like and sometimes dislike. About 68% of students feel that this model has improved their confidence in learning. About 25% of students believe that their confidence in learning has hardly improved. 7% of the students thought the new learning method lowered their confidence. Additionally, some students report that the teacher’s speech expression in class has become smoother. Thus, the new learning method introducing ADL and EC helps improve the enthusiasm in class and the confidence in learning. The students’ experience is excellent. A small number of students believe that failure to improve their enthusiasm and confidence in learning is one aspect of their own receptive ability. The new learning method is still being perfected in some respects.

Academic performance is very critical for every student. Tables 4, 5 compare the results of Group 1 and Group 2.

**Table 4:** Comparison of the academic performance of the ordinary students in the two groups before the experiment.

Group	*N*	Mean value	Standard deviation	Sig.
One	150	87.26	14.698	0.854
Two	150	91.45	11.368	0.806

**Table 5:** Comparison of the academic performance of the ordinary students in the two groups after the experiment.

Group	*N*	Mean value	Standard deviation	Sig.
One	150	98.23	9.456	0.025
Two	150	88.25	12.361	0.0289

In Table, Sig. < 0.05. Thus, there is a significant difference in above-average students’ performance before and after introducing ADL and EC methods. Therefore, introducing ADL and EC has significantly improved the performance of above-average students without much influencing the ordinary and extraordinary students’ academic performance.

### Optimization of English teaching paths in colleges and universities

Students are the future development force of China, and solving the problems in the teaching process is a problem that every educator must face. The corresponding suggestions are put forward from four aspects: (1) continuously update the educational concept of the development of the times. Educators are the main promoters of Ideological and Political Education (IAPE). Their educational philosophy always affects teachers’ in-depth thinking on teaching. It affects every teacher’s thinking on teaching discourse by educational concepts. (2) University educators need richer knowledge literacy and extensive theoretical literacy. Only by accumulating a large amount of English knowledge and practical knowledge can they play an exemplary role. (3) While enriching their own cultural literacy, college teachers should also concentrate on students’ on-class learning situations. Teachers should try their best to enable students to learn, use, and continuously improve students’ EPL. (4) Learn to innovate. Teachers in colleges and universities can often learn new teaching strategies through academic communication and continue to add vitality to the classroom. Introducing ADL into CET can enhance the interaction between students and teachers, thereby improving students’ academic performance.

## Discussion

With the deepening of China’s opening-up, English has become an important communication tool. From the perspective of multiculturalism, this work studies the current situation of CET based on ADL theory. It finds that introducing ADL theory into English classrooms can improve students’ interest in learning English and their self-confidence. Sun et al. (2021) claimed that ADL was an inevitable trend to adapt to social development. With the continuous science, technology, and social program, simple repetition of information could not meet the social development requirements. Wu (2022) believed that DL would be conducive to the reform of education and teaching. The efficiency of DL would be improved by exploring human learning mechanisms and constructing new learning methods. Teaching should focus on learning and creating new knowledge and metacognitive behaviors. Scientific monitoring, adjustment, and evaluation of the learning plan would improve the dilemma of English vocabulary learning through the theoretical guidance of DL. Students would be more confident in the English classroom. DL also gives teachers some thinking on teaching English vocabulary, making teachers focus on cultivating students’ awareness of vocabulary learning and students’ learning strategies (Wu, 2022). The above research theories of the two scholars are similar to the conclusions drawn in this work. They both found that DL was conducive to students’ English learning. The difference is that this work uses comparative analysis to divide students into two groups at random and compares their academic performance over a period of time. The conclusion is reasonable. Additionally, from different perspectives, this work puts forward corresponding suggestions on how to teach college English in the future effectively. The full text is logical and scientific.

## Conclusion

With the continuous socio-economic development, English is playing an increasingly important role. By introducing ADL and EC methods into CET, these conclusions are drawn. (1) The purpose of college students learning English and their time learning English are reviewed, and certain problems are found. Most students’ learning is shallow, remembering vocabulary without understanding. (2) The current teaching situation of college English teachers is analyzed from three vocabulary teaching strategies. Under each strategy, most students have shown perfect acceptance from the teacher’s perspective. However, from students’ perspectives, new teaching strategies must be innovated. (3) Introducing ADL and EC methods into CET has won the approval of most college students. Students believe that the new teaching strategy can make the classroom a more active and harmonious teacher-student relationship. (4) Introducing ADL and EC into CET has enhanced students’ interest in learning and attention on the classroom. The classroom atmosphere is getting active, and students’ homework efficiency is increasing. The academic performance of above-average students is improved. Additionally, most students become confident in learning and are more active. Analyzing the research results shows that introducing DL theory into College English classrooms can not only improve students’ interest in learning. Also, it also enhances students’ self-confidence. Therefore, this work enriches the theoretical research in this field and provides an effective method for CET in the future.

Last but not least, the adopted QS method has certain limitations in data acquisition, resulting in some deviations in the test of relevant data. There is no discussion on the economic investment of DL and EC in CET. The subsequent benefit evaluation can be carried out according to the specific situation, and the suggestions and opinions can better optimize the problems of CET. It is suggested to pay attention to teachers’ and students’ views and concerns and whether their students’ and teachers’ concerns overlap after introducing ADL theory into English teaching.

## Data availability statement

The raw data supporting the conclusions of this article will be made available by the authors, without undue reservation.

## Ethics statement

The studies involving human participants were reviewed and approved by Chengdu University of Technology Ethics Committee. The patients/participants provided their written informed consent to participate in this study. Written informed consent was obtained from the individual(s) for the publication of any potentially identifiable images or data included in this article.

## Author contributions

All authors listed have made a substantial, direct, and intellectual contribution to the work and approved it for publication.

## Funding

This work was supported by the Multicultural Research Center in Sichuan (No. DYWH2006); Philosophy and Social Science Fund of Chengdu University of Technology (No.YJ2021-QN010); National Natural Science Foundation of China (61201438); Key Projects of Sichuan Provincial Department of Education (18ZA0235); Research Fund of Sichuan Province University Key Laboratory of Internet Natural Language Intelligent Processing (INLP201904); and Research Fund of Leshan Normal University (LZD003).

## Conflict of interest

The authors declare that the research was conducted in the absence of any commercial or financial relationships that could be construed as a potential conflict of interest.

## Publisher’s note

All claims expressed in this article are solely those of the authors and do not necessarily represent those of their affiliated organizations, or those of the publisher, the editors and the reviewers. Any product that may be evaluated in this article, or claim that may be made by its manufacturer, is not guaranteed or endorsed by the publisher.
